# Toward identification and intervention to address financial toxicity and unmet health‐related social needs among adolescents and emerging adults with cancer and their caregivers: A cross‐cultural perspective

**DOI:** 10.1002/cam4.7197

**Published:** 2024-04-25

**Authors:** Melissa P. Beauchemin, Samrawit Solomon, Claudia L. Michaels, Kathryn McHenry, Eleanor Turi, Rhea Khurana, Gabriella Sanabria

**Affiliations:** ^1^ School of Nursing Columbia University Irving Medical Center New York New York USA; ^2^ Herbert Irving Comprehensive Cancer Center Columbia University Irving Medical Center New York New York USA; ^3^ School of Medicine Columbia University Irving Medical Center New York New York USA; ^4^ Perelman School of Medicine at the University of Pennsylvania Philadelphia Pennsylvania USA; ^5^ College of Public Health, University of South Florida Tampa Florida USA

**Keywords:** adolescents and young adults (AYA), financial toxicity, health‐related social needs

## Abstract

**Purpose:**

We qualitatively explored the unique needs and preferences for financial toxicity screening and interventions to address financial toxicity among adolescents and emerging adults (younger AYAs: 15–25 years) with cancer and their caregivers.

**Methods:**

We recruited English‐ or Spanish‐speaking younger AYAs who were treated for cancer within the past 2 years and their caregivers. Semi‐structured interviews were conducted to explore preferences for screening and interventional study development to address financial toxicity. The data were coded using conventional content analysis. Codes were reviewed with the study team, and interviews continued until saturation was reached; codes were consolidated into categories and themes during consensus discussions.

**Results:**

We interviewed 17 participants; nine were younger AYAs. Seven of the 17 preferred to speak Spanish. We identified three cross‐cutting themes: *burden*, *support*, and *routine, consistent, and clear*. The burden came in the form of unexpected costs such as transportation to appointments, as well as emotional burdens such as AYAs worrying about how much their family sacrificed for their care or caregivers worrying about the AYA's physical and financial future. Support, in the form of familial, community, healthcare institution, and insurance, was critical to mitigating the effects of financial toxicity in this population. Participants emphasized the importance of meeting individual financial needs by routinely and consistently asking about financial factors and providing clear guidance to navigate these needs.

**Conclusion:**

Younger AYAs and their caregivers experience significant financial challenges and unmet health‐related social needs during cancer treatment and often rely on key supports to alleviate these unmet needs. When developing interventions to mitigate financial toxicity, clinicians and health systems should prioritize clear, consistent, and tailorable approaches to support younger AYA cancer survivors and their families.

## Graphical abstract

Adolescents and emerging adults with cancer and their caregivers experience financial burdens that are often navigated with the support from multiple levels. Participants in our qualitative study offer suggestions to inform future interventions to mitigate the negative impact of the financial costs of cancer care.

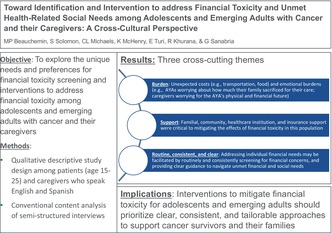

## 1 INTRODUCTION

Adolescents and young adult cancer survivors (AYAs, age 15–39 years) face devastating financial toxicity,^1^, ^2^ defined as the negative personal financial impact of healthcare costs.^3^ Financial toxicity is conceptualized as the combined impact from material hardship, psychological distress, and coping behaviors that an individual patient and their family experience in response to these costs.^4^, ^5^ For AYAs, identified by the National Cancer Institute as a vulnerable population experiencing cancer health disparities,^6^ financial toxicity results from the direct and indirect costs of cancer care, as well as from interrupted education, exclusion from the workforce, and developmental disruption in achieving or maintaining independence.^7^, ^8^ Compared to older cancer survivors, AYAs experience disproportionately higher rates of financial toxicity and unemployment, associated with poorer overall survival and bankruptcy.^2^, ^4^, ^7^, ^9^, ^10^, ^11^ Among AYA survivors in the United States (U.S.), those with public insurance, who live in areas of high deprivation, or who are Black or Hispanic/Latinx (hereafter Hispanic) have lower rates of 5‐ and 10‐year survival,^12^, ^13^, ^14^, ^15^, ^16^, ^17^, ^18^, ^19^, ^20^ likely due in part to adverse social determinants of health (SDOH) and unmet health‐related social needs (HRSN: financial strain, food insecurity, un/underinsurance, unstable housing, and suboptimal education).^20^, ^21^, ^22^ Thus, financial toxicity and HRSN are key contributors to health outcome disparities among AYA survivors.^23^

The risk of financial toxicity is well established for adolescents and emerging adults with cancer (hereafter *younger AYAs* age 15–25 years, inclusive), and recent studies have described preferences among this group that are important to tailor AYA intervention development.^24^, ^25^ Younger AYAs are distinct from the pediatric population because they are developing greater social and financial autonomy,^26^ yet they are more likely than older AYAs and adults to have parental or caregiver involvement in their care decision‐making.^21^, ^27^ The experience of financial toxicity and effective interventions to identify and mitigate it may differ from adult and pediatric populations with cancer; however, less is known about how to effectively screen for and intervene on financial toxicity for younger AYAs and caregivers, including those who speak languages other than English.^28^

To guide the development of financial screening and intervention development to address the unique needs and preferences of younger AYAs and their caregivers, we conducted a qualitative descriptive study^29^, ^30^ among an ethnically and socioeconomically diverse group of younger AYAs with cancer and their caregivers.

## 2 METHODS

### 2.1 Setting

This qualitative descriptive^29^ study was conducted at a large, urban academic center in New York City in the U.S. that cares for a highly diverse racial, ethnic, and socioeconomic population.

### 2.2 Recruitment and consent

Participants were recruited from the outpatient pediatric and medical oncology clinics over a 6‐month period. AYAs age 15–25, inclusive, were eligible if they (1) had undergone treatment for cancer in the past 2 years, (2) were not currently being treated for a recurrence or progression of their cancer, and (3) spoke English or Spanish. Caregivers of the AYAs could be a parent, grandparent, partner, or other significant individual to the AYA; caregivers were eligible to participate if they spoke English or Spanish. AYA and caregiver dyads were encouraged but not required to avoid the unnecessary exclusion of AYA participants without a caregiver to co‐participate. We identified eligible AYAs from an electronic health record‐generated report and purposively sampled^31^, ^32^ across language and role (AYA, caregiver). Potential participants (AYAs and caregivers) were mailed a recruitment letter to introduce the study. For those who did not opt out of future contact, the study team, which included bilingual (English and Spanish) research assistants with qualitative training, followed up to determine interest in participating in a one‐time interview. Interested participants were consented remotely, and an IRB‐approved information sheet was sent via email or text.^33^ We requested that interviews be conducted individually. Participants were each sent a $50 gift card after interview completion. All study procedures were reviewed and approved by the Columbia University Institutional Review Board (AAAT8368).

### 2.3 Study procedures

Interviews were conducted in English or Spanish using Zoom or WhatsApp, per participant preference. Participants completed a brief sociodemographic survey to assess age, race and ethnicity, sex, role (AYA or caregiver), health literacy,^34^ income, level of education, employment status, and insurance type. The interview guide was developed by our study team in consultation with key stakeholders, including an AYA cancer survivor, caregiver advocate, and clinicians with experience caring for AYA patients (Data S1).^35^ The interview guide drew on published frameworks of financial toxicity and burden^5^, ^36^, ^37^ to allow for open‐ended responses from participants regarding how they may or may not have experienced the direct and indirect financial costs of cancer care (i.e., material hardship), how they and their family felt about these costs (i.e., psychological response), and how they adapted or managed these costs (i.e., coping behaviors). The interviewers were trained to probe in depth throughout the session to explore acceptable screening processes to identify financial difficulties and to assess what resources or interventions the AYA and caregiver perceived might reduce financial burdens or address unmet needs.^38^ All study materials were available in English or Spanish. Participants were contacted during the analysis for member checking, a methodologic approach to ensure accuracy and enhanced credibility of results.^39^

### 2.4 Data analysis

Audio recordings of the interviews were transcribed in their language of origin using NVivo Transcription and cleaned to remove participant identifiers. Each transcript was inductively coded by two to three study team members, including bilingual research assistants and the PI, using a conventional content analysis approach.^40^ All interviews were coded in their language of origin to increase trustworthiness and prevent loss of meaning during translation.^41^, ^42^ The interviewer was included in each coding team. The coders held standing meetings with the PI to establish the codebook, compare coding structures and categories, and achieve intercoder agreement. As guided by qualitative descriptive methodology,^29^, ^43^ efforts were made to remain close to the data. Discordance in coding was resolved through team discussions; the team employed a reflexive approach during their coding, both individually and as a team.^44^ After initial coding was completed and saturation was reached across cross‐cutting themes, the coding team reviewed the coded transcripts to explore similarities or differences by participant role and language.^45^ Saturation tables were created and reviewed to ensure agreement between codes and categories.^46^ Interpretations of the data were enhanced through consultation of the notes taken during member‐checking discussions to confirm the findings fit the experiences of the participants.^47^

## 3 RESULTS

Of 51 AYAs who were identified as potential participants and received recruitment material, none opted out from further contact. Of 18 potential participants who our team were able to contact, 17 (nine AYAs and eight caregivers) consented and completed an interview between April and September 2022. These 17 participants represented six dyads, one triad (one AYA and two caregivers), and two AYAs without caregiver participation. One interview included more than 1 participant per AYA preference, and the remaining were completed individually with either the AYA or caregiver. Seven interviews were conducted in Spanish and the remaining 10 in English. All AYA participants were at least 18 years at the time of the interview. Table 1 provides additional demographic information on all participants except one Spanish‐speaking caregiver. Brief exemplars have been included directly in the text, and Tables 2 and 3 provide additional thick descriptive quotations.^39^

**TABLE 1:** Demographic characteristics of interview participants (*n* = 16^a^).

	AYA (*N* = 9)	Caregiver (*N* = 7^a^)
Sex		
Female	4	5
Male	5	2
Race and ethnicity		
Hispanic/Latinx	7	5
Non‐Hispanic white	2	2
Language		
English	6	4
Spanish	3	3
Educational attainment		
≤High school graduate	4	3
Some college or Bachelor's degree	5	4
Employment status^b^		
Employed for wages (part‐time or full time)	4	6
Unemployed for >1 year	2	0
Student or homemaker	2	1
Health insurance coverage		
Yes	8	3
Private health insurance	3	3
Medicaid/SSI	5	0
No	1	4
Health literacy		
High	8	5
Low	1	2
Annual household income		
<$30,000	3	3
>$30,000	1	2
Unknown or declined to report	5	2
Age (years), mean, (range)	21 (18–25)	43.7 (29–51)

^a^ One participant did not complete sociodemographic survey; participant was a caregiver and completed the interview in Spanish.

^b^ Not completed by one participant.

**TABLE 2:** Themes, categories, and exemplars from AYA and caregiver interviews translation to English if interview was conducted in Spanish.

Theme (categories)	Exemplar quote
BURDEN	
Cost burdens	Just the tolls were about $25–30 dollars because we live in Jersey. Then parking was another $25. Then gas, obviously so, I'd say that every time we came in, it costed about 100 bucks. (Caregiver, 3b) I ended up getting like two jobs while I was in treatment…It was definitely hard. There were days where, I couldn't go to work because I was puking my brains out. It took some time management, and my work was really flexible with me. (AYA, 5a) Entonces, en ese momento cuando me dijeron ya es como, en una semana que nos dan una receta, que tendríamos que comprar unas pastillas, que ese frasco de pastillas que contenía como 30 pastillas apenas, que costó casi 700 dólares. Entonces es ahí donde, pues, la verdad es que sí, me preocupó. Dije bueno, pues entonces, pastillas para…a los medicamentos digo. Y ya pues, de aquí para adelante ya, este, yo los tengo que comprar, digo, para que, yo los tengo que, los tengo que comprar, digo. Porque por mi sobrino, digo, porque, porque es algo importante. Digo para mí, es importante comprar los medicamentos. (Caregiver 6b) [*So, in that moment when they told me, it's already like, in a week they're going to give us a prescription, that we'll have to buy some pills, that that bottle of pills contained just 30 pills, which cost almost 700 dollars. So that's where, well, the truth is that yes, I got worried. I said, well then, pills for…I mean medicines. And now well, from now on now, uh, I have to buy them, I mean, so that, I have to, I have to buy them, I mean. Because for my nephew, I mean, because, because it is something important. I say for me, it is important to buy the medicines*.] So suddenly we're getting these very expensive bills for mental health. I told the hospital, “I am sorry, I'm not paying this. I appreciate the value in having a psychologist who specializes in pediatric cancer patients. But this is like out of hand.” We were able to work it out, but there was that. Then there's the health of the rest of the family. I certainly went for counseling. That's like I said, for the vast majority of quality mental health it is generally not covered by insurance. (Caregiver, 3b)
Emotional burdens	The medications, sometimes it was really stressful having to ask my parents or my siblings for money when I couldn't buy medication, mostly because they would just be like, just swallow the pill and I couldn't do it. (AYA, 5a) My child, himself, even though he wants to go to work, he has pain in his legs. I said, you can't go to work, you have to get better, once you get better completely, then you will go to work. But he's like, Mom, I have to help you. I said, I know, you're worried that we're not going to be able to pay for the bills, but somehow, they are going to get paid and don't worry about it. (Caregiver, 2b) Entonces, digo, pero qué tal, digo, si un día, como, a decirle en el hospital que, eh, ya no le pueden dar su tratamiento por falta de por no tener, digamos, este, un seguro que pueda, digamos, cubrir para su tratamiento. Digo pues, ahí sí, como que. Pues sí, la verdad es que a mí me preocupa este, algo por ahí. (Caregiver, 6b) [*So, I mean, but what about, I mean, if one day, like, to tell him at the hospital that, uh, they can no longer give him his treatment due to not having, let's say, um, insurance that can, let's say, cover the cost of his treatment. I say well, there yes, like that. Well yes, the truth is that I worry about this, about something like that*.] As a single mother now of four kids, I know they're adults, two of them are in college and [AYA] with the cancer and the older one is working. So, it's been a… It was a rough year for me because I spent the year with him in the hospital and I didn't work, but the pandemic was going on, and I was furloughed from work. So, it's hard. It's really hard right now for me. (Caregiver, 2b) Sometimes it's just means some things that you would otherwise get done on your own, you don't have the mental capacity to do it because you're exhausted. (Caregiver, 3b)
	My child, himself, even though he wants to go to work, he has pain in his legs. I said, you can't go to work, you have to get better, once you get better completely, then you will go to work. But he's like, Mom, I have to help you. I said, I know, you're worried that we're not going to be able to pay for the bills, but somehow, they are going to get paid and don't worry about it. (Caregiver, 2b) Entonces, digo, pero qué tal, digo, si un día, como, a decirle en el hospital que, eh, ya no le pueden dar su tratamiento por falta de por no tener, digamos, este, un seguro que pueda, digamos, cubrir para su tratamiento. Digo pues, ahí sí, como que. Pues sí, la verdad es que a mí me preocupa este, algo por ahí. (Caregiver, 6b) [*So, I mean, but what about, I mean, if one day, like, to tell him at the hospital that, uh, they can no longer give him his treatment due to not having, let's say, um, insurance that can, let's say, cover the cost of his treatment. I say well, there yes, like that. Well yes, the truth is that I worry about this, about something like that*.] As a single mother now of four kids, I know they're adults, two of them are in college and [AYA] with the cancer and the older one is working. So, it's been a… It was a rough year for me because I spent the year with him in the hospital and I didn't work, but the pandemic was going on, and I was furloughed from work. So, it's hard. It's really hard right now for me. (Caregiver, 2b) Sometimes it's just means some things that you would otherwise get done on your own, you don't have the mental capacity to do it because you're exhausted. (Caregiver, 3b)
SUPPORT	
Family support	Mi mamá y mi papá tomaban las decisiones entre los dos, todas las decisiones. Mi mamá sabía cómo manejar un poco mejor el dinero…y mi papá era que, hacer dinero porque a mi mamá no tenía tiempo para poder trabajar. Pero ahora si tiene tiempo ya. Gracias a Dios. (AYA, 9a) [My mom and dad made the decisions between the two of them, all the decisions. My mom knew how to handle money a little better…and my dad was the one, making money because my mom didn't have time to work. But now she does have time. Thank God.] Yeah, I was supported by my family, by the aid of my parents. I was getting income from my family, I lived with my mom and my sister back then. So, all my financial help was from my family. (AYA, 4a) Here and there, when I would go for my appointments, my dad always told me, just let me know how much it costs. I'll pay you back. When I'm paying for parking or things like that. A lot of times I just quote unquote forgot to remind him and I would just swallow the cost. It's fine, they're doing enough for me. I could pay the $25 for parking. (AYA, 3a) Mi hermano. Es la única persona que tengo a mi lado, porque como soy madre soltera, son tres niños. Bueno, no son niños…el mayor tiene 20. Mi hermano me ha estado ayudando mucho. (Caregiver, 8b) [My brother. He is the only person I have by my side, because since I am a single mother, there are three children. Well, they are not children, the oldest is 20. My brother has been helping me a lot.] Es la salud del niño. La prioridad ahora mismo. (Caregiver 8b) [It's the child's health. The priority right now.] La esperanza y ante todo hay que tener fe, este, en Dios…ha sido fuerte, pero es una experiencia. Pero porque está igual cerca, te da una noticia así, lo que tú buscas es la fe en Dios y todo eso lo pones en la mano de Dios. (AYA, 8a) [The hope and above all you have to have faith, um, in God…it has been hard, but it is an experience. But because it is still close, it gives you news like that, what you are looking for is faith in God and you put all that in God's hand.]
Community support	I remember it being tight at times. The beginning, certainly in the beginning of the first 5 weeks, my brain wasn't even working, and I know we were getting help…I went to work once or twice, maybe. But…I think it was only because I had help from others that I was able to make ends meet. There was no way that would have happened without that help. (Caregiver, 3b) We do have a lot of—where I live—of lot of organizations, all kinds of organizations that help with everything. Then we just had neighbors, friends, relatives help out. Either they helped us with groceries or they sent over stuff like during a holiday or just throughout. Yeah, it was amazing! I was able to complete my Master's without really paying for it and people helped out. Yeah. (AYA, 1a) So, when someone offers to help you do something, the chances are you're going to need it even if you don't think you do. So, don't be so‐ there are some cultures, more than others where taking help from outsiders is like taboo. Take the help, take it, and then there comes a time someone else might be sick. You'll help them pass it on. (Caregiver 3b)
Healthcare Team and Institutional support	I don't think most hospitals, doctors are going to ask you‐ it's not a doctor's job, really. They never, they never did…but I did, through (the social worker), there was an office down the hallway … of the clinic, [they] helped us apply for some scholarship when [my child] was in college. (Caregiver, 1b) I remember this one time I was really financially in the gutter. I couldn't pay for food and my brother wasn't able to because I don't know what happened at home. My sister wasn't able to pay rent well. So, my brother had to cover her side of the rent as well. So, I remember [the social worker] gave me like this $100 gift card from like a place that helps people with food. So yeah, I did some groceries. (AYA, 5a) Yo no he vuelto a trabajar jamás porque me he dedicado a estar con él en su proceso de terapia en el hospital. Ha sido bien, bien difícil. Y la trabajadora social del hospital me ha dado un poco de ayuda, pero para mí y mi familia ha sido bien difícil. (Caregiver, 8b) [I never went back to work because I have dedicated myself to being with him in his therapy process at the hospital. It has been very, very difficult. And the social worker at the hospital has given me a little help, but for me and my family it has been very difficult.]
Insurance support	Todas las medicinas, pastillas, todo lo que [ininteligible] al hospital, lo pagaba mi seguro médico. (AYA, 9a) [All the medicines, pills, everything [unintelligible] the hospital, my health insurance paid for.] There has been some medications that we have had to pay out of pocket. We have to pay co‐payments for the medication. There has been some, but in terms of treatment, the Medicaid has covered most of it. (Caregiver, 4b) I don't know what it would have costed us. It would have been very expensive for us to treat her. Medicaid covered everything and they covered everything given to her, her medications, the doctors, her everything. Her car ride. They had a car service program you could call up. The whole system, how they send you cars, they take you and come pick you up. So that was amazing. (Caregiver, 1b)

**TABLE 3:** Routine, consistent, and clear: Key recommendations to address financial toxicity and unmet HRSN.

Initiating and following up on financial conversations	No, este tipo de conversaciones me han caído bien por el tema de que, nos hacían entender en las cosas que uno tenía que enfocar el tema. Sobre los ahorros de dinero, y, y todo tipo de cosas también. (AYA, 9a) [No, I liked these types of conversations because of the fact that, they made us understand the things that one had to focus on. About saving money, and, and all kinds of things too.] Okay, so I asked one of them, they gave us a printout of a whole booklet of different scholarships you could apply for, after that we were on our own. You don't really get much from that, maybe if you apply to others, but I didn't have time where I had to deal with it… but that would have been nice if they would have been more‐ like the person that I got the form from, maybe she could have followed up on it. There wasn't follow up on that. That would have been nice. (Caregiver, 1b) Knowing that there's someone who can guide them as soon as they get in the process, I think it would set the stage for how they might approach that, whether they will have this defeatist attitude that's going to give up. And whether they're going to give up or not is going to affect how proactive they are, I would imagine, in tending to their needs. (Caregiver, 3b) I was made aware of these programs…by the various PR communication. In other words, I didn't go looking for it. I probably wouldn't have known where to start…Because these organizations actually communicated that these programs are available, I was then able to go ahead and take advantage of those opportunities. (AYA, 8a) Yeah…yeah…because, you know, sometimes you have to like research stuff. And if the information is there that you could just dial a phone number and have someone looking to see if they could help you instead of you researching it. (Caregiver, 2b) I can remember when I was first diagnosed and all of a sudden everyone's introducing themselves and it was a blur. I remember, I came to the clinic for the first time. I wasn't sure who was a doctor, who was a social worker, who was a resident, who was a fellow, who was an attending. It was so much to handle. Like this person was a nurse practitioner in charge of the study. I wasn't sure who to ask my questions to, so definitely not at the beginning. I definitely think things have to settle down. I remember eventually I caught on to the fact that I was going through three‐week cycles and I knew what to expect in terms of how I felt. So, I feel like once I knew what to expect in terms of how I felt and who was who… I know it's not a real time. I think for me, it probably took two months to two and a half months after diagnosed I'd probably when, I was able to start thinking about now that I was diagnosed and now I'm here. (AYA, 3a)
Transparency of roles and responsibility	I guess the social worker is the person that knows the system, that can navigate, that knows the ins and outs. Perhaps someone on the outside, someone with a little more cultural competence. (Caregiver, 3b) At every step, knowing where the hand off is, a very clear handoff to the next person that can help them make that decision. The next step might be a social worker, might be someone in the finance department, might be a doctor, might be clergy, but I would imagine as long as there's a hand off, here's what you're going to do next. There's no like impending doom. (AYA, 7a)

### 3.1 Theme 1: “It was really stressful having to ask my parents or my siblings for money when I couldn't buy medication.” [Burden]

AYAs and caregivers endorsed a variety of burdens related to financial experiences that made their cancer treatment experiences more difficult. Categories within this theme were (1) the financial burden of costs, expected and unexpected, and (2) the resulting emotional burden of financial losses and hardships. Our team considered this theme a confirmation of financial toxicity, encompassing the concrete hardships, the psychological impact, and the response of the patient and family to financial stressors.^5^

#### 3.1.1 Cost burdens

All participants experienced unexpected costs during the AYAs cancer treatment. AYAs and caregivers frequently cited the costs of unmet HRSN,^48^ specifically food, housing, utilities, and transportation. For AYAs and caregivers who were already struggling with finances prior to the cancer diagnosis, they often relied on local resources and community organizations, partnering with social workers or other hospital support networks to provide gift cards or vouchers, or relying on GoFundMe donations. Participants who were financially stable prior to diagnosis expressed shock over the costs of parking at the hospital, commuting, eating on the road, and gas, causing enormous shifts in family budgeting practices. Most participants mentioned housing as a financial challenge; for example, “you know, like whatever they paid in FMLA [*Family Medical Leave of Absence*], it's not even enough to pay a mortgage for one month” (Caregiver 2b).

Costs that were related to cancer treatment and symptom management but were not covered by insurance often presented a burden. Both AYAs and caregivers reported that insurance often would not fully cover oral medications, especially for supportive care, or dietary needs during medical treatment. Similarly, psychosocial health costs (e.g., therapy, counseling, exercise) were frequently reported as difficult or impossible to cover, in contrast to other costs that were covered by insurance. Respondents expressed they were “getting these very expensive bills for mental health” (Caregiver, 3b) and could not pay them, or were upset they could not provide mental health support for the rest of the family. Affording supportive care required time, effort, and navigation through a multitude of people and organizations; some said it was easier to forego the hassle.

Changes in employment led to the unanticipated loss of income for many respondents. Caregivers mentioned the lengthy treatment and chronicity of cancer treatment that hindered their ability to regain their prior financial status and savings. These losses were particularly impactful on AYAs and caregivers working in the gig economy (e.g., food delivery, Lyft/Uber drivers). One AYA reported needing to go back to work even though their family were not supportive of them working because of their fragile health and the risk of exposure (e.g., COVID‐19) and harm (e.g., biking on busy streets to deliver food).

#### 3.1.2 Emotional burdens

Nearly all respondents mentioned the emotional burden that the costs of care caused their families. Some AYAs expressed worry about how much their family had sacrificed for them to receive care, including reducing income, increasing out‐of‐pocket costs, or often, both. AYAs often avoided worrying their family with additional stressors (such as medication and transportation fees) unless absolutely necessary, and that they “would just swallow the cost” (AYA, 3a).

For caregivers, few mentioned a “burden” when discussing the AYA's cancer, but rather expressed enduring worries, including concerns for the well‐being, including financial stability, of the AYA. Concerns included difficulty maintaining insurance for the AYA or worries that the caregiver was failing in other responsibilities (e.g., caring for other family members, their career). To many, however, the most important thing amidst the worries was “la salud” (“the health”) of the AYA.

### 3.2 Theme 2: “Because I had help, I was able to make ends meet.” [Support]

Support in many forms and from many different sources was a major cross‐cutting theme that informs future screening and intervention development, as the participants clearly highlighted the importance of multiple types of support to mitigate financial burdens. AYA and caregivers emphasized the importance of support in their ability to navigate and manage financial burdens and stressful situations such as those mentioned in Theme 1. Support was characterized by four unique categories: (1) family support, (2) community support, (3) healthcare team and institutional support, and (4) insurance support. As one caregiver said, “certainly for someone with less community support or less financial support, less familial support, automatically when finance started, it was like ‘Ahh!’ you know” (Caregiver, 1b).

#### 3.2.1 Family support

Most participants endorsed family financial support as critical to navigating cancer treatment for the AYA. Families often shared responsibilities for financial support evenly, or split duties by having one family member maintain employment and work extra hours while another took on a role as primary caregiver to the AYA. Family definitions varied, sometimes including only those living in the household and other times encompassing extended family. Participants mentioned their *brother‐in‐law* and *uncle* in conversations about who helped them when “tight at times.” AYAs frequently mentioned the desire to be employed to support their family's financial needs. For many participants, a major focus of family financial support was to cover the rent or mortgage.

Participants also described how their families made collective financial decisions. Most reported that finances were discussed as a family to ensure there was a plan to cover costs from the AYA's cancer treatment and related expenses. Most caregivers emphasized that, while finances were discussed with the AYA, they preferred to manage finances, allowing the AYA to focus on cancer treatment. Most AYAs were aware of their family's financial circumstances, a finding similarly endorsed by participating caregivers.

Caregivers often mentioned that family emotional support was critical to managing financial stresses and worries. Some caregivers cited their religion and faith as a support, providing them with hope that all aspects of their AYA's treatment would work out, including the finances. For AYAs, emotional resilience was often tied to their ability to return to work and contribute to the family finances.

#### 3.2.2 Community support

Support from external community organizations and the broader community was often mentioned as important and helpful in covering financial costs: “We do have a lot of‐ where I live‐ a lot of organizations, all kinds of organizations that help with everything… I was able to complete my Master's without really paying for it and people helped out” (AYA, 1a). Common types of community support included grants provided by local or national foundations and community partners, vouchers, or one‐time payments that were critical to paying bills. Few Spanish‐preferring participants mentioned receiving community financial support.

#### 3.2.3 Healthcare team and institutional support

The most common source of financial support within the hospital system was through the social work team. Some mentioned that “the hospital helped” broadly, without specifying through whom or in what form the support came. The lack of support from other members of the healthcare team was not mentioned as a negative. Some caregivers and AYAs stated that the members of the healthcare team outside of social work should focus on clinical care and not be involved in financial support or discussions.

#### 3.2.4 Insurance support

Nearly all participants reported that insurance provided support for covering the costs of care. Some specifically cited the comprehensive coverage and minimal out‐of‐pocket costs of Medicaid, “We have to pay co‐payments for the medication. There has been some, but in terms of treatment, the Medicaid has covered most of it” (Caregiver, 4b). This allowed them to use their income or savings to cover other expenses that they would not otherwise be able to afford.

### 3.3 Theme 3: “I imagine as long as there's a hand off, here's what you're going to do next. There's no impending doom” [Routine, consistent, and clear]

AYAs and caregivers offered suggestions to reduce financial burdens during and after cancer treatment (Table 3). AYAs were often unaware of community resources that provide support until later in their treatment, and they suggested standardizing the process to provide information to all patients and families. The initiation and follow‐up on resource‐related conversations with patients and families were critical to them accessing help: “… because these organizations actually communicated that these programs are available, I was then able to go ahead and take advantage of those opportunities” (AYA, 8a). Some caregivers had received information on resources, but repeated check‐ins with families were recommended because circumstances changed with the loss or gain of employment, the AYA's cancer status or treatment plan, and unanticipated costs.

The optimal process to screen for financial concerns varied, with some saying as soon as possible after diagnosis, whereas others disagreed given the chaotic and overwhelming nature of this time. One AYA recommended that an intervention addressing financial burden should “definitely not [be] at the beginning [of treatment]. I definitely think things have to settle down.” (AYA, 3a). Both AYAs and caregivers said that clear roles, continuity, accountability, and trust would be critical to effective screening for and action to mitigate financial burdens. There were differing opinions regarding whether healthcare providers should be involved in assessing or discussing financial burdens, with some AYAs and caregivers suggesting that the clinical team should focus on cancer treatment. Because of the variability in family structure and responsibility, understanding who is/are involved in financial decision‐making (i.e., parent, another caregiver, AYA) was said to be important to effective screening and intervention success. A few caregivers mentioned that an intervention may be helpful to AYAs if it focused on empowering them to engage with the healthcare system in a more sustainable way to avoid a “defeatist attitude.” Some participants who voiced experiences of significant financial hardships felt like it was them against the hospital and insurance companies, and thus an intervention must be tailorable to individual experiences and financial concerns in the context of a trusting relationship with the person/s or group administering the intervention.

During member checking with AYA participants, we presented our findings and proposed that an intervention that addressed financial toxicity and HRSN by providing tailored referrals to community resources may help to mitigate these burdens and improve the experiences and outcomes of AYA cancer survivors. Respondents to these telephone calls uniformly endorsed this approach.

### 3.4 Comparisons across role and language preference

AYAs and caregivers, regardless of language preference, endorsed a variety of financial challenges and suggested that support in many forms is critical to managing and absorbing these costs of care. Nuances were observed between the AYA and caregivers, and between Spanish and English‐speaking participants.

AYAs were aware of the impact of the cost of their cancer, especially the downstream effects on affording food and housing. Almost universally, they preferred a direct intervention to address these needs, either through existing community support networks or through the direct provision of resources. AYAs were focused on returning to work to financially contribute to the family and regain a sense of agency, even when their family did not yet think they were ready. Caregivers often preferred to shield AYAs from financial worries, even though AYAs were involved in financial discussions.

Among the Spanish‐preferring participants, there was often an emphasis on “familia” (“family”) and, sometimes, on “fe en Dios” (“faith in God”) as a key support to managing financial stress. When discussing support, Spanish‐preferring participants named an individual, often another family member or a social worker. In contrast, English‐speaking participants additionally mentioned institutional support as a means to address their financial needs.

## 4 DISCUSSION

Our qualitative study connects the financial burdens of cancer to the strategies and support that are often relied on to manage these stressors and provides guidance for financial screening and intervention development for culturally and linguistically diverse younger AYAs with cancer and their caregivers. As research continues to expand our understanding between financial toxicity, adverse determinants of health, and resulting unmet HRSN, our qualitative inductive study design and inclusion of participants preferring English or Spanish allow further insight into the importance of support from the family, community, healthcare team, and insurance companies. Thus, this study offers suggestions to effectively screen for and address financial toxicity among diverse younger AYAs with cancer and their families. Financial screening and navigation interventions should consider integrating accessible resources and trusted support systems to meet the unique needs of an AYA and, where appropriate, the caregiver and family.

Three cross‐cutting themes emerged from the data: burden, support, and the importance of an intervention that routinely provides clear and consistent support to address the financial challenges experienced by AYAs and their caregivers. Our study confirmed the extensive financial hardships that AYAs and their families experience during cancer treatment, both indirect and direct, the negative psychological impact, and the coping responses to manage these stressors.^4^, ^5^, ^10^, ^36^ Additionally, as Danhauer et al. and Nightingale et al. both reported, the financial experiences of AYA cancer survivors are closely tied to the support of their family or caregiver and should be considered in the socioecological context of their family, care team, health system, and state or federal policies.^10^, ^49^ Our study offers a closer examination of the experiences and preferences for financial screening and support among a diverse group of AYAs and caregivers. This expands our understanding of the type of support needed to address not only financial toxicity but the closely tied health‐related social needs that were so common among our study participants.

Though distinct, the financial and psychological burdens were often intertwined among the AYAs and caregivers in our study. AYA cancer survivors have high psychological distress compared to younger and older survivors of cancer, and promotion of well‐being is increasingly recognized as important to improving health outcomes among AYAs.^50^ Most AYAs and caregivers endorsed the importance of emotional support, including help from family, community, institutions, and religion, to maintaining resilience and managing financial stresses during a difficult time. Our data support the importance of routine and consistent screening for unmet needs,^51^ and for interventions to include tailored support to meet the needs of the AYA, such as psychosocial, vocational, or educational needs, depending on their personal goals and life stage.^50^, ^52^, ^53^, ^54^

In our study, factors related to insurance or medical bills were less frequently associated with financial burden, in contrast with other studies.^1^, ^55^, ^56^ AYAs in our study were all insured with either Medicaid or parental insurance coverage, which may protect against financial toxicity.^57^ Additionally, this study was conducted in a Medicaid‐expansion state, a factor associated with improved access to insurance and cancer outcomes.^58^, ^59^ The insurance experiences of AYAs in other states or in other countries may differ and warrant further investigation. AYAs in our study may also have benefited from organizational processes commonly supported at academic medical centers, such as prior authorizations and copay assistance. This is an example of an organization‐level intervention^60^, ^61^ that may be effective in limiting or reducing financial toxicity for younger AYAs.

Our analysis highlighted nuanced experiential differences between participants. Interventions to address financial toxicity and HRSN needs should include standardized components but also be tailorable depending on the AYA's age, reported HRSN, and other preferences, such as personal navigation or case management support compared to self‐directed digital navigation of available resources.^62^ Caregivers expressed more anxiety and worry about finances and the AYA's financial future. This supports the need for a different support intervention compared to the more pragmatic concerns of AYAs, such as financial education or insurance literacy training interventions that may be directed to the AYA, the caregiver, or to a family unit.^28^, ^63^ Additionally, cultural context and humility should guide the development and implementation of financial screening or needs navigation.^64^, ^65^ A more holistic assessment of personal preferences may lead to more acceptable and effective interventions to address individual AYA and caregiver social needs. Indeed, most participants in our study were able to navigate complicated financial situations due to support from multiple levels; future interventions should strive to facilitate needs navigation, support AYAs and caregivers, and reduce financial distress. Effective interventions should be clear regarding the roles of involved individuals and systems to promote transparency, accountability, and trust‐building between AYAs, caregivers, and the healthcare team.^66^

### 4.1 Strengths/limitations

We used rigorous qualitative research methods; however, our study has limitations. Interviews were conducted and transcribed in two different languages. To avoid potential inconsistencies, we used conventional qualitative content analysis, followed best practice guidance for multilingual qualitative research,^42^ and held routine team meetings to discuss codebook development and ongoing analysis. Additionally, our sample size was small, due primarily to a protracted study period, and this limited our ability to conduct further dyadic analysis and formally compare AYA and caregiver experiences.^67^ This may lead to limited transferability;^39^ however, we reached saturation during coding procedures across all participants. Our study is strengthened by the representativeness of Spanish‐preferring participants because the majority of financial toxicity research has excluded this group of AYAs. Therefore, our findings, which largely align with prior research on financial burden among AYAs and caregivers, offer nuance to guide financial toxicity screening procedures and intervention development for Spanish‐preferring younger AYAs.^10^, ^49^ Our study reflects, at least on a broader level, multicultural populations.

## 5 CONCLUSION

In conclusion, our qualitative study of AYAs and caregivers confirms the extensive negative financial experiences during cancer treatment, yet highlights the creative and necessary leveraging of supports to navigate financial challenges. These findings will guide research to adapt or develop novel screening strategies and a tailorable intervention, to address financial toxicity for a diverse population of younger AYAs with cancer and their caregivers.

## AUTHOR CONTRIBUTIONS

**Melissa P. Beauchemin:** Conceptualization (lead); data curation (equal); formal analysis (lead); funding acquisition (lead); investigation (lead); methodology (lead); project administration (equal); resources (lead); software (lead); supervision (lead); validation (equal); visualization (equal); writing – original draft (equal); writing – review and editing (lead). **Samrawit Solomon:** Conceptualization (supporting); data curation (supporting); formal analysis (equal); project administration (equal); writing – original draft (lead); writing – review and editing (supporting). **Claudia L. Michaels:** Formal analysis (equal); methodology (equal); writing – review and editing (equal). **Kathryn McHenry:** Formal analysis (equal); methodology (supporting); visualization (supporting); writing – original draft (supporting); writing – review and editing (supporting). **Eleanor Turi:** Formal analysis (supporting); writing – review and editing (supporting). **Rhea Khurana:** Project administration (supporting); writing – review and editing (supporting). **Gabriella Sanabria:** Formal analysis (equal); writing – review and editing (equal).

## FUNDING INFORMATION

Columbia Nursing Intramural Pilot Award (MB).

## CONFLICT OF INTEREST STATEMENT

The authors have no conflicts of interest to disclose.

## ETHICS STATEMENT

All study procedures were reviewed and approved by the Columbia University Institutional Review Board (AAAT8368).

## Supporting information


Data S1.

Data S2.

## Data Availability

Qualitative data are available from the authors upon reasonable request. The codes utilized during the current study are available from the authors upon reasonable request.
