# Supplementary material for: Toward identification and intervention to address financial toxicity and unmet health‐related social needs among adolescents and emerging adults with cancer and their caregivers: A cross‐cultural perspective
Source: Cancer Med. 2024 Apr 25;13(8):e7197. doi: 10.1002/cam4.7197 (PMC11043682; doi:10.1002/cam4.7197)
Supplement: Supplementary file 1 — Data S1. [file CAM4-13-e7197-s002.pdf]

Thank you for taking the time to speak with me today. As we discussed during the consenting process, the reason for this interview is to learn about how cancer *has affected you and your family's financial situation. We want to understand what it is like for adolescents and young adults with cancer and their families to pay for things, such as medications, healthcare, food, and other things so that we, as researchers and healthcare providers, can help others going through similar experiences in the future.*

I am going to ask you some questions and may follow-up on your responses either to explore something further or to be sure I understand what you mean. There are no wrong or right answers, and I encourage you to be honest and share your experiences and feelings. You may also decide to not answer some or any of my questions. The interview should last between 20 and 40 minutes, and, in addition to recording the interview, I may write some notes down while you talk.

Before we get started, I would like to collect sociodemographic information, I will read the questions aloud to you. You may also decide to not answer any of the questions.

(Go to REDCap and complete sociodemographic questionnaire in the record number corresponding to the participant's signed consent form)

Do you have any questions before we get started? (once answered, begin audiorecording)

1. **First, how are you feeling today? (in particular for the AYA patient but also for partner)**
    a. **(for AYA only) When were you diagnosed with cancer, and what treatment are you currently receiving or did you receive?**
    b. Throughout your treatment, were you ever admitted inpatient for a portion of your treatment?
    c. Have you had any unplanned emergency department visits?

2. **Thinking back to before you (your child/family member) were diagnosed with cancer, can you tell me a bit about how you and your family paid for things, like housing, food, or clothing?**
    a. Can you tell me more about how these decisions were made and who was involved in making these decisions?
    b. How did you and your family make these decisions?
        i. *AYA specific*: Were you responsible for any other financial costs in the year before your cancer diagnosis?
        ii. How did the pandemic effect how you and your family paid for these things?
            1. Did the pandemic change how you made decisions about money? If yes, how did this change?

3. **Now, since you've been diagnosed with cancer, can you tell me about how it has been like for you and your family *as it relates to money and your ability to pay for the things you and your family need since you* (your child/family member) were diagnosed with cancer?**
    a. How have your needs changed?
    b. How have you and your family decided what to prioritize?
    c. How was this different from before the cancer diagnosis?
    d. Have you used any programs or resources to help with cancer costs or household costs?

4. **What have been the most difficult costs or money issues that have come up since you (your child/family member) were diagnosed with cancer?**
    a. *(direct costs)*: Have there been specific costs that you have had to deal with since the diagnosis? What costs did you expect/did not expect to deal with after the diagnosis?

Columbia University IRB
IRB-AAAT8368 (Y01M04)
IRB Approval Date: 09/20/2022
For use until modified or study is closed

i. How have you/your family responded to this experience?

b. *(indirect costs):* Have you or anyone in your household had to stop working or has your family's income decreased due to cancer treatment?
    i. If yes, who stopped working? Was there a discussion about who would stop working? Can you tell me more about that?

5. **Have you experienced any worry/stress/anxiety about the costs related to your cancer care?**
    a. If yes:
        i. What were the costs that caused you worry?
        ii. Were you able to resolve these worries and cover the costs? If yes, can you tell me how you did this?
        iii. What were the most unexpected costs related to cancer?
        iv. What worries you the most about your financial situation?

6. **(Coping) Were there ever times where you or someone in your family had to make adjustments to usual routines to cope with the financial costs of cancer treatment?**
    a. How were other family members affected by these changes in your usual routine?
    b. Were you (your AYA) unable to make scheduled doctor appointments or take medications that you needed due to financial concerns? How about other members of your family? (e.g., spouse, parent, other children)

7. **Do you remember someone from the healthcare team asking you if you had any concerns about money or your financial situation?**
    a. Can you tell me when this occurred and who was in the room when this happened? Was it helpful?
        i. If yes, how was it helpful?
        ii. If no, how could have gone better and been more helpful?
    b. How often did these conversations take place and where did they take place? Were you ever provided with information (through conversation, reading material, etc.) about the potential costs that may come up during cancer treatment?
        i. If yes, did you find this information helpful?
            1. How did this information make you feel?
    c. How comfortable or willing are you to discuss your financial situation in general?
        i. If not willing or uncomfortable, how about if doing so could provide you with resources or extra financial support?
        ii. Who would you feel most comfortable discussing your finances with?
            1. *Probe:* someone from the healthcare team? A social worker? Someone within the hospital but outside of your direct healthcare team? Someone outside of the hospital entirely, like an external organization for cancer patients?

8. **We've heard from other adolescents/young adults that they sometimes have different concerns or experiences with money compared to younger kids or older patients that they may meet or see in clinic/hospital.**
    a. Do you agree with that statement?
    b. If not, can you tell me why you think the experiences are not different?
    c. If yes, can you tell me about your own unique experience?
        i. If participant brings up any of the above, probe into how they experienced these concerns: fertility or reproductive concerns, sexual health, education, early career, young parenting concerns

Columbia University IRB
IRB-AAAT8368 (Y01M04)
IRB Approval Date: 09/20/2022
For use until modified or study is closed

d. (*For the caregiver)* What financial information do you think is appropriate and important to share with adolescents and young adults about the costs related to their care?
    i. Have the ways you discuss household finances changed since the AYAs healthcare diagnosis?
    ii. Do you think there are any benefits or negative consequences to an open conversation about finances in improving the AYAs financial education?

9. **In your experience, what are the most important financial or money-related concerns for adolescents and young adults with cancer?**
    a. Can you think of something that would be helpful for other individuals and their families? When do you think is it appropriate to address these concerns (immediate after the diagnosis and later)?
    b. What, if anything, has been most helpful for you with regard to finances?

10. **(Optional: if the interview is very long and the participant seems anxious to end, this can be skipped) I'd like to hear about a silver lining, a positive experience, or outlook you have gained since the cancer diagnosis?**
    a. *(Probe, if quiet or no ideas*) We've heard from other participants that they have done something (with their family) that was unexpected, or they hadn't done before. Have you had an experience like that?

(If nothing else to say) – Thank you for your time and for sharing your experiences and thoughts today. We know that these discussions can be difficult, and we appreciate that you shared with us. Can I ask if we can reach out to you in the future when we are putting together the results of these interviews, to ensure that our interpretation of what you said sounds correct to you?

Again, thank you so much for your time, and we will send you the link for your gift card shortly. Take good care

Columbia University IRB
IRB-AAAT8368 (Y01M04)
IRB Approval Date: 09/20/2022
For use until modified or study is closed
