# Supplementary material for: Toward identification and intervention to address financial toxicity and unmet health‐related social needs among adolescents and emerging adults with cancer and their caregivers: A cross‐cultural perspective
Source: Cancer Med. 2024 Apr 25;13(8):e7197. doi: 10.1002/cam4.7197 (PMC11043682; doi:10.1002/cam4.7197)
Supplement: Supplementary file 2 — Data S2. [file CAM4-13-e7197-s001.pdf]

Gracias por tomarse el tiempo de conversar conmigo hoy. Como hemos comentado durante el proceso de consentimiento, el motive de esta entrevista es conocer cómo el cáncer ha afectado a su situación económica y a la de su familia, así como a su capacidad de pagar las cosas. Queremos entender cómo es para los adolescentes y jóvenes adultos con cáncer y sus familias el pago de cosas, como los medicamentos, la atención sanitaria, la comida y otras cosas que nosotros, como investigadores y proveedores de atención sanitaria, podemos ayudar a otros que pasen por experiencias similares en el futuro.

Voy a hacerle algunas preguntas y puede que siga sus respuestas, ya sea para explorar algo más o para estar seguro de que entiendo lo que quiere decir. No hay respuestas correctas o incorrectas, y le animo a que sea sincero y comparta sus experiencias y sentimientos. También puede decidir no responder a alguna o algunas de mis preguntas. La entrevista debe durar entre 45 y 60 minutos y, además de grabarla, es posible que tome algunas notas mientras usted habla.

¿Tiene alguna pregunta antes de empezar? (una vez contestada, comienza la grabación de audio)

1. **En primer lugar, ¿cómo se siente hoy? (en particular para el paciente AYA, pero también para la pareja)**
    a. **(sólo para AYA) ¿Cuándo le diagnosticaron el cáncer y qué tratamiento está recibiendo actualmente?**

2. **Si pensamos en el pasado, antes de que a usted (a su hijo o a un miembro de su familia) le diagnosticaran cáncer, ¿podría contarme cómo pagaban usted y su familia cosas como la vivienda, la comida o la ropa?**
    a. ¿Puede decirme más sobre cómo se tomaron estas decisiones y quiénes participaron en el proceso de tomar decisiones?
    b. ¿Cómo tomaron usted y su familia estas decisiones?
        i. *Específicamente AYA:* ¿Fue usted responsable de algún otro gasto financiero en el año antes de su diagnosis de cáncer?
        ii. ¿Cómo afectó la pandemia a la forma en que usted y su familia pagaron estas cosas?
            1. ¿La pandemia cambió su forma de tomar decisiones sobre el dinero? En caso afirmativo, ¿cómo cambió?

3. **Ahora, ¿podría contarme cómo ha sido para usted y su familia con respecto al dinero y a su capacidad para pagar las cosas que usted y su familia necesitan desde que a usted (su hijo/familiar) le diagnosticaron cáncer?**
    a. ¿Cómo han cambiado sus necesidades?
    b. ¿Cómo han decidido usted y su familia a qué dar prioridad?
    c. ¿Cómo se diferenciaba esto de antes del diagnosis del cáncer?

4. **¿Cuáles han sido los gastos o problemas de dinero más difíciles que han surgido desde que a usted (su hijo/familiar) le diagnosticaron cáncer?**
    a. *(gastos directos)*: ¿Hubo gastos específicos a los que tuvo que enfrentarse desde el diagnóstico? ¿Qué gastos esperaba/no esperaba tener que asumir tras el diagnóstico?

Columbia University IRB
IRB-AAAT8368 (Y01M02)
IRB Approval Date: 05/19/2022
For use until modified or study is closed

        i. ¿Cómo ha respondido usted o su familia a esta experiencia?

   b. (*gastos indirectos*): ¿Usted o alguien de su familia ha tenido que dejar de trabajar o han disminuido los ingresos de su familia debido al tratamiento del cáncer?
        i. En caso afirmativo, ¿quién dejó de trabajar? ¿Hubo una discusión sobre quién dejaría de trabajar? ¿Puede decirme algo más al respecto?

5. **¿Ha experimentado alguna preocupación por los gastos relacionados con el manejo de su cáncer?**
   a. En caso afirmativo:
        i. ¿Cuáles fueron los gastos que le preocuparon?
        ii. ¿Pudo resolver estas preocupaciones y cubrir los costes? En caso afirmativo, ¿puede decirme cómo lo hizo?
        iii. ¿Cuáles fueron los gastos más inesperados relacionados con el cáncer?
        iv. ¿Cuál es el aspecto que más le preocupa de su situación financiera?

6. **(Afrontamiento) ¿Hubo ocasiones en las que usted o alguien de su familia tuvo que hacer ajustes en las rutinas habituales para afrontar los costes financieros del tratamiento del cáncer?**
   a. ¿Cómo afectaron a otros miembros de la familia estos cambios en su rutina habitual?
   b. ¿Se vio usted (su AYA) incapaz de acudir a las citas médicas programadas o de tomar medicamentos que necesitaba debido a problemas económicos? ¿Y otros miembros de su familia? (por ejemplo, el cónyuge, los padres, otros hijos)?

7. **¿Recuerda si alguien del equipo sanitario le preguntó si tenía alguna preocupación sobre el dinero o su situación financiera?**
   a. ¿Puede decirme cuándo ocurrió esto y quién estaba en la sala cuando sucedió? ¿Fue útil?
        i. En caso afirmativo, ¿en qué le ha servido?
        ii. Si no, ¿cómo podría haber ido mejor y haber sido más útil?
        iii. ¿Con qué frecuencia sucedían estas conversaciones y dónde tenían lugar?
   b. ¿Se le proporcionó alguna vez información (en conversaciones, material de lectura, etc.) sobre los posibles costes que pueden surgir durante el tratamiento del cáncer?
        i. En caso afirmativo, ¿le ha resultado útil esta información?
            1. ¿Cómo le hizo sentir esta información?

8. **Hemos escuchado de otros adolescentes/jóvenes adultos que a veces tienen diferentes preocupaciones o experiencias con el dinero en comparación con los niños más pequeños o los pacientes mayores que pueden conocer o ver en la clínica/hospital.**
   a. ¿Está de acuerdo con esta afirmación?
   b. Si no es así, ¿puede decirme por qué cree que las experiencias no son diferentes?
   c. En caso afirmativo, ¿puede hablarme de su propia experiencia?
        i. Si el participante menciona alguno de los puntos anteriores, investigue cómo ha experimentado estas preocupaciones: preocupaciones sobre la

Columbia University IRB
IRB-AAAT8368 (Y01M02)
IRB Approval Date: 05/19/2022
For use until modified or study is closed

fertilidad o la reproducción, salud sexual, educación, carrera profesional temprana, preocupaciones sobre la crianza de los hijos

9. **En su experiencia, ¿cuáles son las preocupaciones financieras o monetarias más importantes para los adolescentes y jóvenes adultos con cáncer?**
    a. ¿Puede pensar en algo que sería útil para otras personas y sus familias? ¿Cuándo cree que es apropiado abordar estas preocupaciones (inmediatamente después del diagnóstico y más tarde)?
    b. ¿Qué es lo que más le ha ayudado, si es que hay algo, con respecto a las finanzas?

10. **Me gustaría escuchar una experiencia positiva que usted (y su familia) hayan tenido desde el diagnóstico de cáncer.**
    a. *(Si está callado o no tiene ideas)* Hemos escuchado de otros participantes que han hecho algo (con su familia) que fue inesperado o que no habían hecho antes. ¿Has tenido una experiencia así?

(Si no hay nada más que decir) – Gracias por su tiempo y por compartir sus experiencias y pensamientos hoy. Sabemos que estas discusiones pueden ser difíciles, y le agradecemos que las haya compartido con nosotros. ¿Puedo preguntarle si podemos ponernos en contacto con usted en el futuro cuando recopilemos los resultados de estas entrevistas, para asegurarnos de que nuestra interpretación de lo que ha dicho le parece correcta?

Otra vez, muchas gracias por su tiempo, y le enviaremos el enlace para su tarjeta de regalo enseguida. Cuídese mucho.

Columbia University IRB
IRB-AAAT8368 (Y01M02)
IRB Approval Date: 05/19/2022
For use until modified or study is closed
